# Male Hormonal Contraception—Current Stage of Knowledge

**DOI:** 10.3390/jcm14072188

**Published:** 2025-03-23

**Authors:** Julia Bania, Joanna Wrona, Kacper Fudali, Franciszek Stęga, Piotr Filip Rębisz, Marek Murawski

**Affiliations:** 1 Faculty of Medicine, Wroclaw Medical University, 50-367 Wroclaw, Poland; joanna.wrona@student.umw.edu.pl (J.W.); kacper.fudali@student.umw.edu.pl (K.F.); franciszek.stega@student.umw.edu.pl (F.S.); piotr.rebisz@student.umw.edu.pl (P.F.R.); 2 Clinical Department of Gynecologic Surgery and Oncology, Wroclaw Medical University, Borowska 213, 50-556 Wroclaw, Poland; marek.murawski@umw.edu.pl

**Keywords:** male hormonal contraception, male contraception, contraception, DMAU, 11β-MNTDC, Nestorone, MENT, spermatogenesis, spermatogenic rebound

## Abstract

Male hormonal contraception has been the focus of extensive research efforts aimed at identifying effective and reversible methods for male fertility control. This review summarizes the current state of knowledge, key achievements, and future directions in the development of male hormonal contraception. A review was conducted using the PubMed, Embase, and Scopus databases. The search strategy included terms such as “male hormonal contraception”, “Nestorone”, “7α,11β-Dimethyl-19-nortestosterone 17β-undecanoate (DMAU)” and “11β-methyl-19-nortestosterone 17β-dodecylcarbonate (11β-MNTDC)”. A total of 107 references were analyzed to synthesize the most relevant findings regarding the hormonal contraceptive agents under investigation. The review outlines historical and recent advancements in male hormonal contraception, highlighting compounds that have demonstrated limitations in effectiveness, side effects, or inconvenient administration. Notable candidates under study include 7α-methyl-19-nortestosterone (MENT), DMAU, 11β-MNTDC, and the combination of segesterone acetate with testosterone in gel form. These agents show promise due to their ability to suppress follicle-stimulating hormone (FSH) and luteinizing hormone (LH), leading to effective spermatogenesis inhibition with minimal side effects. Additionally, the phenomenon of spermatogenic rebound is considered. Among the investigated agents, oral DMAU, 11β-MNTDC, and the Nestorone–testosterone gel appear to be the most promising candidates for male hormonal contraception due to their high efficacy, user-friendly administration, and favorable safety profiles. However, further large-scale clinical trials are necessary to confirm their long-term effects on human health and fertility, ensuring their viability as future contraceptive options.

## 1. Introduction

Despite the wide availability and variety of contraceptive methods on the market, modern reversible contraceptive options for men have garnered global interest [1]. The results of a 2022 survey indicate that as many as 75% of men in the USA and Canada are interested in using hormonal contraception for men [2]. Consequently, the market for such contraception methods, counted using these data, is estimated between 7 million and 15.5 million potential users [2]. Equally important to note is the positive attitude of women in this matter [1]. In fact, depending on the survey, between 42.8 and 94% of women would be interested in using potential male contraception as a couple [1]. This highlights the widespread need for such contraceptives, which would allow for greater equity in family planning and more effective management of shared risk in couples when the woman has contraindications to oral contraception or is experiencing side effects [3]. Currently, condoms remain the only widely accessible and reversible contraceptive method for men [4]. What is more, 14% of men worldwide declare the use of condoms [5]. However, their effectiveness in preventing pregnancy, with a failure rate of 13%, is not as satisfactory compared with the methods available for women (intrauterine contraceptive devices (IUDs) have a failure rate of 1%) [6]. Condoms’ effectiveness is strongly dependent on correct use, during which frequent user errors or condom dysfunction appears [7]. Condoms are also indicated as a cause of erection issues in between 5.3 and 28.1% of men [7]. Additionally, between 7 and 44.7% of condom users experience problems with fit and feel [7]. The undeniable advantage of using condoms is its effectiveness in preventing the transmission of many sexually transmitted diseases [8].

Vasectomy is expensive for individuals and often requires state funding, which remains scarcely available [9]. In 2022 in a study of Chinese men, vasectomy reversal surgery was successful in 160 of the 242 patients studied [10]. This makes it perceived as a permanent contraception method [11]. Consequently, the frequency of its use is strongly dependent on culture of origin, education, and wealth; nevertheless, its median prevalence remains at 6% in the USA [11] and 2.3% worldwide [5]. However, it remains an extremely effective option, with a failure rate of under 1% [12]. Attention should also be paid to possible complications of vasectomy associated with surgical interference, the most common of which are infection (1.22%) and hematoma (1.56%) [12]. Post-vasectomy pain syndrome occurs even less frequently, in 0.14% of patients [12]. It is also important to acknowledge that there is usually up to a three-month delay in the effectiveness of vasectomy. The effectiveness must be verified by the sperm count [13]. The above data regarding effectiveness, safety, and social acceptance of condoms and vasectomy are compared in Table 1.

Consequently, the burden of contraception in heterosexual relationships is often disproportionately placed on women; furthermore, the methods above are frequently overlooked by healthcare providers when offering advice on the most appropriate contraception options [14]. Male hormonal contraception emerges as a promising alternative in this context, demonstrating efficacy comparable to female hormonal contraceptives, which operate by a similar mechanism of action [4].

**Figure 1:**
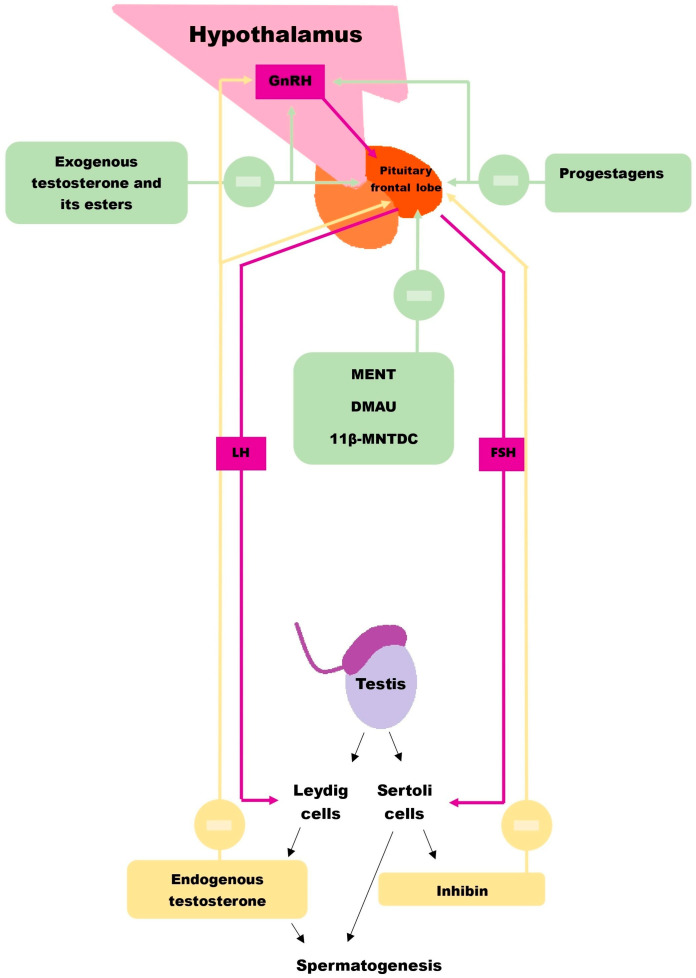
The feedback loop of male sex hormones is illustrated. They are crucial in the process of spermatogenesis. Tropic hormones are marked in purple, hormones produced within the testes are marked in yellow, and substances investigated for male hormonal contraception are marked in green. Arrows with the shape of circles with a rectangle in the center indicate an inhibitory effect, while arrows without this shape signify the stimulation. The figure also depicts the target sites of action for compounds studied as potential male hormonal contraceptives. DMAU—7α,11β-dimethyl-19-nortestosterone 17β-undecanoate, 11β-MNTDC—11β-methyl-19-nortestosterone 17β-dodecylcarbonate, MENT—7α-methyl-19-nortestosterone, LH—luteinizing hormone, FSH—follicle-stimulating hormone, GnRH—gonadotropin-releasing hormone.

The effectiveness of hormonal contraceptive agents lies in reducing the spermatozoid concentration in semen to below 1 million spermatozoids/mL [15]. It is achieved by suppressing the hypothalamic–pituitary–gonadal axis and, consequently, lowering intratesticular androgen levels [16]. Physiologically, androgen concentration in the testes is 40 to 100 times higher than in the serum, which is essential for maintaining adequate spermatogenesis [16]. Administering exogenous androgens or their derivatives suppresses the production of gonadotropin-releasing hormone (GnRH), thereby significantly reducing the pituitary secretion of luteinizing hormone (LH), which stimulates Leydig cells to produce testosterone in the testes, and follicle-stimulating hormone (FSH), which, along with testosterone, promotes the Sertoli cell function essential for spermatogenesis (Figure 1) [17]. Using exogenous forms of testosterone in this process avoids the loss of secondary sexual characteristics. However, since the concentration of testosterone in the serum required to suppress spermatogenesis significantly exceeds physiological levels, there is an increased risk of adverse effects associated with its excess, such as reduced high-density lipoprotein (HDL) cholesterol, weight gain, and erythrocytosis [18,19].

Given the broad social demand for male hormonal contraception, as well as the drawbacks of the currently used methods, our research group acknowledge that a thorough synthesis of existing research is both essential and highly relevant. This review aims to synthesize current findings, identify gaps in knowledge, and provide a foundation for future research. By consolidating the available data, our research group seeks to inform healthcare professionals and researchers, ultimately contributing to the development of safe, reliable, and reversible male contraceptive options. The goal of research in hormonal male contraception is to develop a reversible agent that optimally suppresses the hypothalamic–pituitary–gonadal axis while minimizing negative effects on other tissues and organs [20].

## 2. Methodology

A comprehensive literature search was conducted to identify relevant studies for this narrative review. The databases PubMed, Embase, and Scopus were systematically queried using the following keywords: “male hormonal contraception”, “spermatogenesis suppression”, “contraception prevalence”, “contraception safety”, “vasectomy safety”, “contraception risk”, “male contraception attitude”, “testosterone esters”, “testosterone enanthate”, “testosterone undecanoate”, “testosterone buciclate”, “cardiovascular risk”, “GnRH agonists”, “GnRH antagonists”, “androgens and progestins”, “17-α-hydroxyprogesterone”, “medroxyprogesterone acetate”, ”MPA”, “19-nortestosterone”, “NETE”, “levonorgestrel”, “LNG”, “etonogestrel”, “desogestrel”, “7α-methyl-19-nortestosterone”, “MENT”, “NES”, “NES + T”, “nestorone”, “7α,11β-dimethyl-19-nortestosterone 17β-undecanoate”, “DMAU”, “7α,11β-dimethyl-19-nortestosterone”, “DMA”, “11β-methyl-19-nortestosterone 17β-dodecylcarbonate”, “11β-MNTDC”, “11β-methyl-19-nortestosterone”, “11β-MNT”, and “UGT2B17”. These terms were combined using the Boolean operators “OR” and “AND” to ensure a comprehensive search strategy.

Following the screening process, 107 studies were deemed relevant and included in the bibliography. Data extracted from original research articles, narrative reviews, books, and systematic reviews have been considered in the analysis. Only articles published in English have been included in this review.

## 3. Testosterone Esters

Research on male hormonal contraception began in the United States in the 1970s, with an initial focus on testosterone (T) [21]. The rationale for this approach was its ability to suppress gonadotropin secretion via the negative feedback mechanism of the hypothalamic–pituitary–gonadal axis. However, the pure form of testosterone proved unsuitable due to the significant first-pass effect observed with both oral and intramuscular administration. Additionally, the doses required to overcome rapid metabolism were cost-prohibitive for potential distribution and, with the sustained hyperphysiological serum testosterone levels, were associated with side effects such as hypertension, weight gain, mood swings, changes in libido, and polycythemia [22].

To address these issues, testosterone enanthate (TE), a short-acting testosterone ester, was introduced for intramuscular injections. It was already used in the treatment of male hypogonadism [23]. TE was initially studied in small groups of volunteers who were interested in undergoing vasectomy, but had not performed it yet, at Planned Parenthood clinics. Injections were administered at doses of 200 mg weekly or biweekly to achieve oligospermia below 5 million spermatozoids/mL (later revised to 3 million/mL) or azoospermia over several months [23].

Preliminary results were promising in both dosing regimens. Most volunteers who completed the studies achieved the targeted levels of oligospermia or azoospermia, with low rates of pregnancies occurring during subsequent trials. The process was reversible, as participants regained fertility post-treatment. Reported side effects included acne of varying severity, weight gain, injection site pain, and changes in cholesterol and triglyceride levels [23].

In 1990, the World Health Organization (WHO) launched a large-scale, multicenter study involving 271 healthy and fertile men across 10 centers worldwide, including Beijing, Edinburgh, Melbourne, and Stockholm [24]. Each participant received weekly intramuscular injections of 200 mg TE. Among them, 157 (65%) achieved azoospermia within six months and entered the suppression phase, during which spermatogenesis was effectively suppressed while using alternative contraceptive methods. They then proceeded to a 12-month efficacy phase, relying solely on TE as contraception. Only one pregnancy occurred during this phase, despite confirmed azoospermia in the semen samples of the male partner. During the suppression and efficacy phases, 15 participants withdrew due to injection-related discomfort or complications and 27 due to side effects. Adverse effects included weight gain, increased hemoglobin and serum testosterone levels, reduced testicular volume, decreased serum urea, reduced LH and FSH levels, and decreased HDL cholesterol levels. These changes were similar across both azoospermic and non-azoospermic groups, with hormone levels gradually returning to baseline after discontinuation. Fertility was restored in all participants who completed the recovery phase, with spermatozoid concentrations returning to 20 million/mL or higher. Notably, azoospermia rates varied between the centers, reaching 91% in Chinese centers and 60% in non-Asian centers [24]. This difference between varying ethnicities was repeated in another study. According to the latter article of World Health Organization Task Force on Methods for the Regulation of Male Fertility from April 1996, the percent of participants that achieved azoospermia differed between Asian and non-Asian centers: 85.7% and 67.8%, respectively [25]. In addition to its adverse effects, TE is a short-acting testosterone ester with a terminal half-life of 4,5 days [26], which requires weekly intramuscular injections that are associated with tenderness/discomfort at the injection site. Therefore, TE contraception is unlikely to be chosen by the majority of recipients. Moreover, the period necessary to reach significant sperm production suppression took from several months to one year [25]. To conclude, TE has not been adopted in monotherapy for male contraception due to its limitations [27].

Further subsequent research focused on long-acting testosterone esters, particularly testosterone undecanoate (TU) and testosterone buciclate (TB) [28,29]. A single intramuscular injection of 1200 mg TB demonstrated promising results, with azoospermia achieved in 3 out of 8 participants over 32 weeks of follow-up without hyperphysiological testosterone levels. Adverse effects were documented, such as reduced testicular volume as well as significant increases in erythrocyte count, hematocrit, and mean hemoglobin within the notional values. Discomfort at the injection site was denied [30]. TB’s longer dosing interval, due to its half-life of 29.5 days [26], was an advantage, but economic constraints led to its discontinuation [30].

Research on TU, a long-acting T ester with a terminal half-life of 33.9 days [26], began in the 1970s, paralleling the studies on TE [27], and progressed significantly in China [18,29,31]. In a phase III trial, 1045 participants received monthly intramuscular injections of 250 mg TU; 845 participants achieved azoospermia within 6 months and entered the efficacy phase [31], during which 733 completed follow-ups. In summary, 95.2% of participants achieved azoospermia (or severe oligozoospermia) within the suppression phase [31]. Nine pregnancies occurred over 1554.1 person-years, yielding a failure rate of 4.8% due to inadequate suppression and 1.3% due to spermatogenic rebound. Spermatogenesis recovered in all participants, except 17 patients, within 12 months of discontinuation [31].

The detected side effects were transient and included tenderness or discomfort at the injection sites, acne, severe coughing lasting minutes after injection, mood changes, behavioral changes, facial swelling, skin rash, changes in libido (mostly increased), increased body weight, reduced total testis volume, increased mean hemoglobin level, and reduced mean of total cholesterol and LDL cholesterol levels. HDL level changes varied based on ethnicity [18,31]. Values of some of the parameters, such as: testis volume and hemoglobin levels, did not return to their baseline by the end of the study [31]. The above data regarding TE, TB, and TU are compared in Table 2.

Research connected to testosterone esters as a male contraception method proved that there are some differences in response between Asian and non-Asian men in trials. This aspect would be particularly important when inventing a globally universal formula. Cases of non-responders to TE or TB injections in non-Asian participants were reported, while in contrast, non-responders to TE or TU injections were comparably rare in Chinese men [18]. The ethnicity impact is more distinctive when androgen is used alone in the regime, where weekly injections of TE resulted in azoospermia in 91% of Chinese men and 60% of non-Asian men. Once subjects reached azoospermia, its consistency was easier to maintain among Chinese men (95% compared with 68%). In addition, side effects of varying changes within the levels of lipids and liver transaminases were different between the East Asians and Non-Asians [32]. An analysis by Ilani et al. suggested that Asian race is an independent predictor of eventual suppression of sperm output during contraception and faster recovery of the output after discontinuation. On the contrary, Caucasian race can act as a predictor of faster suppression. Mechanisms of the phenomenon might be connected to differences in testicular histomorphometry as well as a suggested lower 5α-reductase enzyme activity in the East Asian men. To achieve adequate suppression of gonadotropins and sperm concentrations among both groups, the addition of progestin is necessary [32].

Ultimately, research on testosterone esters as monotherapy waned with the emergence of more effective and universally applicable agents, such as GnRH agonists and antagonists, progestins, and synthetic androgens. Challenges with oral administration and mixed patient reception of intramuscular injections, along with side effects, further contributed to this decline. Nevertheless, studies on testosterone esters laid the groundwork for the development of hormonal male contraception and informed combination therapy strategies [33].

## 4. Combinations of Androgens and Progestogens

Currently, a crucial aspect of research into male hormonal contraception is the combination of androgens with progestogens. This combination allows for faster and more potent suppression of FSH and LH release, facilitating the achievement of contraceptive gonadotropin levels below <1 IU/L [34,35]. This approach leads to reduced spermatogenesis intensity, lowering spermatozoid concentrations to <1 million spermatozoids/mL in semen, thereby inducing azoospermia or oligospermia [34,35,36]. The effect is particularly significant for Caucasian men, who exhibit weaker responses to testosterone-based contraception alone compared with men of Asian descent [35].

Moreover, compared with testosterone monotherapy, progestogens combined with androgens have been shown to induce apoptosis in the spermatogenic epithelium, not only during androgen-sensitive stages of the spermatogenesis cycle (stages VII–VIII) but also during the early (I–IV) and late (XII–XIV) stages [37]. It is also worth mentioning that exogenously administered androgen prevents the loss of secondary sexual characteristics (physiologically maintained by testosterone and its metabolites), which could otherwise result from the negative feedback loop mechanism triggered by progestogen administration. Progestogen-only administration would lead to a reduction in FSH and LH synthesis and, consequently, a decrease in endogenous testosterone production [38].

Studies have demonstrated that progestogens may act not only on the hypothalamus and pituitary gland but also directly on the testes and epididymis at the gonadal level, which could further enhance the efficacy of progestogens as agents used in hormonal contraception [39]. Studies investigating the effects of male hormonal contraception have consistently demonstrated that combinations of testosterone or its derivatives with progestogens achieve superior results in inducing azoospermia or severe oligospermia compared with testosterone monotherapy. Moreover, these combinations exhibit minimal side effects, presenting promising prospects for the future development of effective male contraceptive methods [40]. Combining progestogens with androgens enables the use of testosterone components at lower doses, thereby reducing the risk of severe androgen-related side effects [41]. Additionally, progestogens could mitigate the adverse side effects of testosterone and its derivatives when used as monotherapy [41]. A consistency between most studies testing contraception based on testosterone are reports of small increases in hematocrit and decreases in HDL levels [42]. Progestogens, specifically, derivatives of 19-nortestosterone or levonorgestrel, when administered in combination with testosterone or its derivatives, may induce side effects similar to those observed with androgen monotherapy. This is due to their agonistic activity at the androgen receptor [41]. However, despite this agonistic effect, progestogens like NETE exhibit lower androgenic activity compared with testosterone [43]. In newer progestogens such as Desogestrel, the androgenic activity is significantly reduced [43]. Other progestogens, such as cyproterone acetate (CPA), exhibit inherent anti-androgenic activity, which could be advantageous in male hormonal contraception. However, studies have shown that when combined with testosterone, CPA yields inconsistent results or leads to undesirable reductions in libido and sexual potency. Therefore, further research is required [43]. Modern studies also focus on identifying the minimum effective dose of a testosterone and progestogen combination required to achieve azoospermia or severe oligospermia while minimizing potential undesirable side effects [40].

Simultaneously, due to their mechanism of action, comparing the effects of progestogens to GnRH antagonists remains challenging. However, GnRH antagonists may be considered a potential alternative to progestogens, as they do not induce the undesirable side effects associated with the addition of progestogens in hormonal male contraception. [39,42]. The administration of progestogens, both with and without testosterone or its derivatives, remains an area of ongoing investigation. The potential side effects of such therapy are not yet fully understood, raising concerns about whether the use of progestogens would be truly beneficial, the safety of this contraceptive approach across different age groups, and which testosterone and progestogen combination would be most effective in achieving a safe and widely accessible male contraceptive method. In any case, further development in this field requires long-term studies [42].

## 5. Derivatives of 17-α-Hydroxyprogesterone

Medroxyprogesterone acetate (MPA) is a synthetic derivative of progesterone that has been used in the treatment of pathological conditions and female contraception [41]. Experiments examining the effects of MPA are predominantly conducted using depot medroxyprogesterone acetate (DMPA), a long-acting derivative of MPA [44]. Intramuscularly administered DMPA combined with TE has been shown to reduce spermatogenesis to azoospermia levels, both at higher doses (250 mg TE + 200 mg DMPA) and lower doses (100 mg TE + 100 mg DMPA) [45].

The optimal frequency of DMPA administration in combination with testosterone injections has also been determined to maximize its effectiveness [41]. In this context, DMPA combined with testosterone was tested over a period exceeding four months in studies involving two groups of men. In the first group, the combination was administered monthly. In the second group, the study period was divided into two phases: an initial phase with higher doses (20 mg MPA + 100 mg testosterone) followed by a phase of gradual dose reduction (20 mg MPA + 50 mg testosterone). Results indicated that spermatogenesis suppression was more pronounced in the group where doses were gradually reduced, with faster achievement of azoospermia. In the first group, 40 out of 85 men achieved azoospermia, while in the second group, 35 out of 46 men achieved azoospermia before the study’s conclusion [41].

MPA has been tested in both oral and subcutaneous injection forms, with subcutaneous administration proving more effective than oral administration [44]. Compared with studies on MPA combined with testosterone, its esters (TU and TE), and 5α-dihydrotestosterone (DHT), the combination with DHT did not exhibit as strong spermatogenesis-inhibiting properties as the combinations with testosterone or its esters. While LH levels among the tested men remained identical, FSH levels in groups receiving DHT were higher (decrease in FSH levels from 6.4 mIU/mL to 3.6 mIU/mL) compared with groups receiving testosterone or TU (decrease from 6.4 mIU/mL to 1.9 mIU/mL and 1.2–2.6 mIU/mL, respectively), which was reflected in spermatozoid concentrations relative to FSH levels [44].

When tested on men of Asian descent, MPA allowed for the achievement of azoospermia or oligospermia below 1 million spermatozoids/mL lasting for three months without severe side effects warranting discontinuation of therapy [44]. Notable side effects included weight gain, often associated with reduced HDL levels in the blood. However, the impact of MPA on the increased risk of cardiovascular diseases has not been studied [45].

## 6. Derivatives of 19-Nortestosterone

19-nortestosterone and its derivatives represent one of the newest areas of research in male hormonal contraception. In the past, these substances were used successfully as a part of hormonal contraception in women [41]. These anabolic steroids exhibit high potency and activity by binding to both androgen and progesterone receptors, producing effects comparable to those of progesterone. However, their use is associated with various adverse effects, including decreased libido and potency as well as increased risk of cardiovascular diseases and, in men, gynecomastia [41,46]. The most notable representatives of this group include norethisterone and its derivatives, norethisterone acetate and norethisterone enanthate.

Norethisterone was the first developed synthetic progestogen to bind to the progesterone receptor. Products containing this compound can be administered orally, subcutaneously via injection, intrauterine, or vaginally [41]. Its unique properties include partial conversion to ethinylestradiol (EE) in the liver, which may contribute to systemic estrogenic effects. Furthermore, EE formation enhances the effects of norethisterone [46]. The mechanism of action of norethisterone also includes binding to androgen receptors, which, when used in female hormonal contraception, results in undesired androgenic effects [43]. When used as a part of male hormonal contraception, norethisterone binds to the progestogen receptor, suppressing FSH and LH secretion via negative feedback loop, thereby reducing endogenous testosterone levels. This effect does not cause side effects associated with testosterone deficiency due to norethisterone’s androgenic activity, which compensates for the endogenous testosterone deficit [43].

In a 2001 study involving 14 men, the combination of norethisterone enanthate (NETE) and TU achieved azoospermia in all but 1 participant [43]. The spermatogenesis suppression results were comparable to those observed in studies involving GnRH antagonists. However, norethisterone acetate offers the advantage of requiring administration only once every six weeks, unlike GnRH antagonists, which require daily dosing [43].

The effects of NETE and norethisterone acetate, which bind to similar receptors, were compared [47]. At identical doses, NETE achieved lower FSH levels than norethisterone acetate, suggesting better gonadotropin suppression by NETE. LH levels did not show significant changes [47].

Side effects of norethisterone use include an increased risk of cardiovascular diseases caused by EE and elevated LDL cholesterol levels [46]. The WHO conducted extensive research on the efficacy of NETE combined with TU as a potential element of male hormonal contraception. However, despite promising results (93% of participants achieved the desired spermatozoid concentration range), the study was discontinued due to severe side effects, including significant mood swings and one participant’s suicide, although it has been stated by the participant’s family that the suicide was due to reasons uninvolved with the administration of the researched substance [48].

Norethisterone, as a derivative of testosterone and an anabolic steroid, may pose potential risks when administered in higher doses [49]. Among these, the increased risk of various cardiovascular diseases is of significant concern, especially in the context of prolonged therapy [49]. This issue has not been thoroughly analyzed, but studies have shown that higher doses of anabolic steroids may increase the risk of hypertrophic myopathy, as demonstrated in animal models [50]. It is also possible that prolonged use of anabolic steroids may raise blood pressure levels [51]. It is possible that progestogens contribute to an increased risk of cardiovascular diseases. Combinations of progestogens and testosterone have been shown to elevate LDL cholesterol, decrease HDL cholesterol, and cause weight gain in men during treatment. Additionally, these combinations lead to an increase in pro-coagulant substances in plasma [42,52], all of which are risk factors for the development of atherosclerosis [42]. While it is true that norethisterone and its derivatives have an agonistic effect on the androgen receptor, their androgenic activity is relatively low. Another notable side effect is the development of a proinflammatory profile, which was observed in a 2005 study involving men receiving testosterone undecanoate (TU) and norethisterone (NETE). This proinflammatory response could potentially increase the risk of cardiovascular diseases due to elevated levels of IL-6. The results of the study led the researchers to conclude that patients treated with these combinations should be closely monitored for signs of inflammatory processes [53]. Regardless, further long-term clinical trials are needed to clearly determine whether the changes induced by progestogens have clinical significance [42].

7α-methyl-19-nortestosterone (MENT) is a synthetic androgen and a potential element of male hormonal contraception that is covered more thoroughly in [54]. It has been demonstrated that MENT can lead to elevated blood pressure, with a notable increase in systolic pressure and a subclinical rise in diastolic pressure. A 2003 study investigating the effects of MENT implants in male patients observed a significant increase in systolic blood pressure among those receiving MENT, with an average rise of 4.8 mm Hg during the first six months of the trial.

In this study, two patients exhibited an exceptionally large increase in blood pressure. Both had an initial reading of 140/90 mm Hg; however, during treatment, their systolic blood pressure rose substantially. One patient recorded a systolic pressure of 150 mm Hg on days 30, 60, 90, and 180 of the study. The second patient, whose blood pressure levels were significantly higher than the study’s mean, had to discontinue participation due to severe hypertension. On day 30, this patient recorded a blood pressure of 160/100 mm Hg, followed by 160/90 mm Hg a week later, and 140/100 mm Hg at the next visit. Due to these elevated readings, the decision was made to remove the MENT implant and exclude the patient from further participation.

Furthermore, the study found that MENT implant treatment resulted in increased hemoglobin, hematocrit, and erythrocyte levels; however, these values remained within their normal physiological ranges [55].

A long-term study from 2007 also demonstrated a small but consistent increase in systolic blood pressure among patients who received either implants containing etonogestrel and MENT or implants containing etonogestrel along with subcutaneous testosterone pellets. Throughout the study, patients in the etonogestrel + MENT group experienced elevated systolic blood pressure levels, while the diastolic blood pressure remained largely unchanged.

Compared with the second group, men administered MENT exhibited higher systolic blood pressure, which could represent a significant risk factor for cardiovascular diseases. Additionally, the treatment led to a slight decrease in HDL cholesterol levels in both groups [54].

## 7. Levonorgestrel

Levonorgestrel (LNG), an isomer of norgestrel, is currently used in intrauterine release systems for female hormonal contraception. LNG is a progestogen that, through binding to the progestogen receptor and negative feedback mechanisms, is one of the most effective and active compounds for inhibiting gonadotropin synthesis [56]. The side effects of LNG use are similar to those caused by other 19-nortestosterone derivatives [41,46,56].

In the body, LNG binds to the androgen receptor, leading to a decrease in sex hormone-binding globulin (SHBG) levels. This results in reduced testosterone binding to SHBG. LNG can be administered in various forms, including orally and via subcutaneous injection [37]. Studies have shown that TE combined with LNG reduces FSH and LH levels in men. Additionally, LNG may inhibit 5α-reductase, directly affecting testicular metabolism by blocking the conversion of testosterone to DHT [56].

Depending on the route of administration, the efficacy of tested substances varies significantly. Oral LNG combined with TE injections resulted in a 36% efficacy increase compared with oral LNG combined with oral testosterone [41]. Promisingly, LNG can be administered via subcutaneous implants, as demonstrated in a 2002 study that examined the effect of the Norplant II implant on spermatogenesis [57]. Daily administration of 500 mg LNG combined with 100 mg TE injections induced oligospermia in 94% of participants, whereas testosterone monotherapy in the form of a transdermal patch achieved desired results in 64% of participants.

In a group where transdermal testosterone patch administration was delayed by three weeks after LNG administration, FSH and LH levels decreased only due to the subcutaneously implanted Norplant II system containing four 160 mg LNG capsules (at week 3, serum LH level 2.92 ± 0.78 IU/L; FSH, 1.69 ± 0.35 IU/L). After administering the testosterone patch, additional reductions in FSH and LH levels were observed (week 6, LH 1.65 ± 0.58 IU/L; FSH, 0.75 ± 0.25 IU/L). FSH levels were lower in the group using the Norplant II implant combined with the testosterone patch compared with the group using the testosterone patch alone. In the third group, which used the Norplant II implant with TE, serum FSH concentrations decreased to less than 0.5 IU/L, maintaining this level from week 3 to week 24. Lowering the LNG dose to 125 mg while maintaining the same TE doses yielded similar results, reducing the frequency of side effects such as weight gain and increased HDL levels [57].

The study demonstrated similar LNG bioavailability in both sexes. The implant combined with the testosterone patch achieved oligospermia below 1 million spermatozoids/mL or azoospermia in 60% of participants, while the implant combined with testosterone injections achieved similar oligospermia in all participants without side effects [57].

## 8. Desogestrel

Desogestrel (DSG) is a prodrug converted in the body to its active form, etonogestrel, which binds to progestogen receptors. DSG, like LNG, can be administered orally but achieves the best results when delivered subcutaneously alongside testosterone injections [58].

In a 2005 study involving 130 men, the combination of subcutaneous testosterone decanoate with a DSG subcutaneous implant achieved oligospermia or azoospermia, maintaining these results for 48 weeks without side effects that would prevent further research. This indicates that DSG could contribute to the development of safe, long-term male hormonal contraception [59].

## 9. MENT

7α-methyl-19-nortestosterone (MENT) is a synthetic androgen whose effect is based on inhibiting gonadotropin synthesis by binding to both androgen receptors and progestogen receptors [60]. What is more, its androgenic activity causes a decrease in endogenous testosterone [61]. MENT does not interact with SHBG [62]. It cannot be administered orally because of inactivation in the digestive tract, so to obtain the appropriate amount in the body, it is necessary to supply MENT in the form of a gel, transdermal patch, or subcutaneous implant [62]. The best effects have been achieved by administering MENT via intramuscular injections containing 7α-methyl-19-nortestosterone acetate (MENTAc), which in vivo undergoes active hydrolysis to the biologically active form, MENT [61]. MENTAc showed greater bioavailability than MENT, suggesting that it is the preferred form of administration of this agent [61].

At the same time, MENT maintains the proper functioning of other androgen-dependent processes within the body without adversely affecting muscle function and bone structure [62]. In men with hypogonadism, MENT was an effective form of androgen replacement therapy.

Studies examining the functionality of MENT in male hormonal contraception conducted in humans did not produce entirely satisfactory results. Spermatogenesis suppression in the studied men differed significantly between participants with long-term use of 135 mg MENTAc together with etonogestrel for contraception [62]. It has been estimated that the increase in FSH levels, accompanied by a slight rise in LH levels due to a possible reduction in sensitivity to MENT with prolonged use (an effect that MENT cannot prevent during long-term administration), was still sufficient to induce spermatogenesis.

Additional side effects of long-term use of 7α-methyl-19-nortestosterone included increased hemoglobin levels, increased hematocrit, significant mood swings, and sporadic increases in HDL cholesterol levels in the blood of the subjects [54]. A potentially hepatotoxic effect of MENT has also been demonstrated. After the administration of 10 mg/kg/day, the retention of clearance of intravenously administered bromsulfthalein was increased. Enhanced levels of alanine aminotransferase, aspartate aminotransferase, γ-glutamyl transpeptidase, and sorbitol dehydrogenase were also detected, which could indicate a potential hepatotoxic effect. However, the increase in the levels of these substances was not significant, and the risk of hepatotoxicity was lower than in the case of administration of 17α-methyltestosterone [63].

MENT is still being considered as a possible compound for use in male contraception, but it requires further research [16]. The research conducted on MENT has been used in the creation and development of newer compounds, 7α,11β-dimethyl-19-nortestosterone (DMA) and 11β-methyl-19-nortestosterone (11β-MNT) [19].

## 10. Nestorone

One of the directions of research on male hormonal contraception is the combination of testosterone with segesterone acetate, which, used in this combination in the form of a gel, would effectively inhibit spermatogenesis and cause a reduced spermatozoid count in the semen [19,38]. Nestorone (NES) is a progestogen derived from 19-norprogesterone, which does not exhibit androgenic, estrogenic, or glucocorticoid activity, which means that side effects of NES may be lower [38]. A characteristic of NES is its low bioavailability after oral administration, yet it is well absorbed when applied percutaneously [64].

Tests have shown that the use of exogenous progestogens such as Nestorone suppresses gonadotropin secretion in men even more effectively and allows for physiological androgen dosing, significantly eliminating hyperandrogenic side effects and minimizing the time to reach effective contraceptive thresholds (<1 million spermatozoids/mL of semen) [65]. The following question raises whether the combination of NES with testosterone will be more effective in providing potent hormonal contraception.

A study conducted by the Los Angeles and Seattle Health Centers focused on assessing the effectiveness of gonadotropin suppression when NES is used alone and in combination with testosterone gel [66]. Nestorone was tested on healthy volunteers to develop a transdermal NES gel together with T gel (gel containing testosterone) as a method of male contraception that is user-controlled and supplier-independent. The decision was made to study the effects of NES and NES + T on gonadotropins before studying their effects on spermatogenesis to determine the effective dose of NES for suppressing gonadotropin secretion and the additive effect when administered with a selected dose of T gel. Studies have shown that men using transdermal NES gel had significantly reduced LH and FSH levels. It is worth mentioning that the decrease in FSH and LH levels in men during hormonal contraception is usually reversible upon discontinuation of the contraceptive. After discontinuation of hormonal medication, the hormonal system usually returns to its natural state and, as a result, physiological spermatozoid production and reproductive function resume [66]. Subjects using NES gel alone experienced a decrease in gonadotropin levels, resulting in significantly decreased serum T levels and reported decreases in libido. Increasing the dose of NES in combination with T resulted in proportional increases in serum NES levels, leading to greater suppression of both gonadotropins. With higher doses of NES (6 or 8 mg/day) in combination with T (10 g/a day) gel, many of the subjects had undetectable serum LH and FSH levels. The assessment of gonadotropin suppression was performed approximately 24 h after gel application, which may affect the range of the negative feedback effects of T and NES. These data provide evidence that although NES has a weak to moderate effect on gonadotropin secretion in men when combined with T, the additive effect is significant [66,67]. The above data are presented in Table 3.

The study, conducted by the Los Angeles Biomedical Research Institute at Harbor-UCLA Medical Center (LA BioMed), Los Angeles, CA, and the Center for Research in Reproduction and Contraception at the University of Washington, Seattle, WA, among men aged 18 to 50 years, was designed to demonstrate the efficacy of testosterone (T) gel combined with the nonandrogenic progestin NES gel, administered transdermally, to maintain constant plasma concentrations of testosterone in suppressing spermatogenesis. The study found that more than 60% of those using NES gel at a dose of 8 mg/day combined with testosterone gel at dose of 10 g/day achieved low spermatozoid concentrations by 8 weeks, and 89% achieved effective contraceptive concentrations (≤1 million/mL) by 20–24 weeks, with no improvement in results achieved by increasing the NES dose to 12 mg/day. Azoospermia occurred in 78% of those using T + NES 8 mg/day and 69% of those using T + NES 12 mg/day. Temporary increases in semen volume happened in several men after the use of these preparations [68]. Furthermore, studies on the efficacy of hormonal prototypes of male contraceptives have shown that reducing semen concentration to ≤1 million/mL (regardless of mobility) is sufficient to prevent pregnancy in the female partner, with efficacy rates equal to or better than the typical failure rates of contraceptive pills approved for women [16,69]. The above data are presented in Table 4.

The conclusions drawn from these studies support the use of transdermal NES in combination with T gel, as it significantly increases the efficacy of suppressing gonadotropins and spermatogenesis [68,70]. NES doses of 8 mg daily combined with 10 g T gel appears to be the optimal dosing regimen, with no improvement in spermatogenesis suppression observed with the higher dose of NES at 12 mg/day.

Another study conducted by the Contraceptive Clinical Trial Network, the University of Washington Center for Research in Reproduction and Contraception, Seattle, WA, and the Center for Men’s Health, Los Angeles Biomedical Research Institute at Harbor-UCLA Medical Center, Los Angeles, CA, examining the simultaneous use of two gels in combination: testosterone (T) gel 10 g + placebo Nestorone (NES) gel (T + NES 0); T gel 10 g + 8 mg NES gel (T + NES 8); T gel 10 g + 12 mg NES gel (T + NES 12). The study examined the efficacy of these combinations in suppressing spermatogenesis [71]. Serum testosterone levels were unrelated to the failure to suppress spermatogenesis at any time point during the study, in contrast to Nestorone levels, which were significantly associated with spermatogenesis suppression [71].

Another study evaluating the efficacy of combinations of testosterone and progesterone derivatives compared four different substances, levonorgestrel, cyproterone acetate, norethisterone acetate (all administered orally), and the progestogen Nestorone^®^, applied transdermally in combination with transdermal exogenous testosterone. The strongest suppression of FSH and LH levels was observed in patients who used cyproterone acetate or levonorgestrel in combination with transdermal testosterone. It is worth noting that progesterone significantly reduces gonadotropin levels, and this effect is intensified by transdermal testosterone administration (LH < 0.5 IU/L, FSH < 0.5 IU/L). The authors emphasize the need for further studies to determine the minimum effective dose of progesterone, which could reduce the incidence of adverse effects [72].

The association between serum NES levels and the suppression of spermatogenesis indicates the importance of adding progestogens to a hormonal contraceptive regimen, which is supported by previous observations and by drug compliance as a predictor of overall success in a hormonal male contraceptive regimen [68].

A study conducted by the Contraceptive Clinical Trials Network among men aged 18–50 years evaluated the efficacy of daily use of a single, combined Nes–T gel containing 8.3 mg Nes and 62.5 mg T compared with 62.7 mg T gel alone in reducing serum FSH and LH levels to ≤ 1.0 IU/L, considered effective for male contraception, and to compare the pharmacokinetics of serum Nes and T levels between the gel groups [73]. The results showed that the Nes–T gel was more effective than the T gel alone in reducing gonadotropin levels. Furthermore, almost 85% of Nes–T users had lower FSH and LH levels than T gel users, an important variable for the efficacy of hormonal contraception in men. After Nes–T use, mean serum gonadotropin levels remained significantly low for 2 days, suggesting that missing one or two doses may not affect the contraceptive efficacy of this gel combination [73].

The conducted studies clearly illustrate the clinical consequences of using transdermal gels. In the aforementioned study [68], patients applied transdermal gels daily for 24 weeks, and adherence to the treatment regimen was monitored by counting empty testosterone sachets, measuring the weight of Nestorone gel containers, and conducting blood hormone level tests. If Nestorone was not detected in a participant’s serum, it indicated non-adherence to the prescribed regimen [68].

This method was relatively simple and non-invasive, as the gels were applied to the skin on the arms and abdomen, eliminating the need for medical visits required for hormonal injections. Most men tolerated the formulations well, and libido as well as sexual function remained stable, contributing to the acceptability of this contraceptive approach [68].

Despite these advantages, some participants faced challenges in maintaining regular gel application. Six men failed to adhere to the regimen to an extent that could allow their data to be included in the efficacy analysis. The high frequency of follow-up visits—18 over 6 months—may have been a burden for participants. Some also expressed concerns about accidental gel transfer to their partners, prompting instructions to avoid skin contact after application or to wear protective clothing. Side effects such as acne, slight weight gain, and mood changes were reported, though they were not severe enough to significantly impact adherence [68].

In summary, the majority of participants adhered to the recommended gel application guidelines, and the method was generally perceived as convenient and potentially acceptable. However, challenges with consistent use highlight the need for further research on improving treatment adherence. Possible solutions include reducing the frequency of follow-up visits or combining both active substances into a single gel to simplify application. Additionally, patient education on proper use and the potential consequences of irregular application could play a crucial role. Further development of formulations that minimize the risk of accidental transfer to others may also be beneficial. Future studies should consider the long-term effects of this therapy and its overall impact on users’ health to provide a more comprehensive assessment of its suitability as an effective and practical method of hormonal contraception for men.

Despite promising results, this method of contraception was not without side effects. The use of two gels, NES and testosterone, was associated with a risk of acne in one-fifth of the study participants, and several participants reported mood swings, changes in sexual function, or low mood or depression [68]. Other reasons for discontinuation included symptoms of asthma exacerbation or insomnia among the study participants [4].

Weight gain was observed in all treatment groups using two gels, one with NES and the other with T, but was not associated with the dose of NES (8 mg/day or 12 mg/day) [68]. HDL cholesterol levels and the HDL/LDL cholesterol ratio decreased during treatment in all T + NES groups, but more so in the higher-dose NES group (12 mg/day vs. 8 mg/day) [68]. In the higher-dose NES group, there was a small but significant increase in fasting glucose levels [68], which supports studies suggesting a diminishing effect of progestogens on insulin sensitivity [74]. When using a gel containing both NES and testosterone, adverse events included decreased libido, sunburn at the application site, and dry, scaly rash [68].

Differences in response to the male hormonal contraceptive regimen may be attributed to biological, genetic, and adherence-related factors [71]. The study shows that serum gonadotropin levels after 4 weeks of treatment strongly predict the efficacy of the therapy, with men who fail to suppress gonadotropins to ≤ 1 IU/L having lower chances of success [71]. However, even adequate gonadotropin suppression does not guarantee full spermatogenesis suppression. Serum LH levels emerged as the key predictor of efficacy, although other factors, such as genotype and adherence to the regimen, also play a role. Furthermore, the addition of progestogen (NES) to the regimen enhances its effectiveness, with adherence to the treatment regimen being critical for success [71]. Another study showed that in hormonal contraceptive trials, about 5–10% of men fail to fully suppress spermatogenesis to ≤ 1 million spermatozoids/mL [38]. While the cause is unclear, persistent intratesticular testosterone and/or FSH/LH levels may contribute. Studies suggest Asian men suppress spermatogenesis more consistently than non-Asian men of European descent, though the reasons remain unknown. Possible factors include testicular histomorphometry, testosterone metabolism, androgen receptor polymorphisms, and gonadotropin suppressibility [38].

Male hormonal contraception in the form of a gel is undoubtedly a future idea that has significant potential to change the way men can actively participate in family planning and birth control [64]. This innovative method offers certain advantages, such as ease of application, which may make it attractive to men looking for effective and easy-to-use contraceptive options that are also safe for their health.

## 11. DMAU and 11β-MNTDC

17β-undecanoate 7α,11β-dimethyl-19-nortestosterone (DMAU) and 11β-methyl-19-nortestosterone dodecylcarbonate (11β-MNTDC) are two substances that predispose to the title of agents used for male hormonal contraception. Both reversibly inhibit the secretion of FSH and LH [63,75,76,77,78].

DMAU is a prodrug metabolized in the body to the active form—7α,11β-dimethyl-19-nortestosterone (DMA). The advantage of DMA is its effectiveness in binding, with a high affinity to human androgen and progestogen receptors [79]. Additionally, as tested in castrated rat models, after subcutaneous administration of a single dose of DMAU, its active form DMA exhibits androgenic activity and leads to LH suppression.

Furthermore, DMAU after oral administration still shows activity in biological tests [79]. The oral route of administration is a major advantage of this substance, because an oral tablet taken once a day is the most desirable form of contraception for men [80]. In 2014, the first study evaluating the pharmacokinetics of DMAU after oral administration in the form of powder capsules in healthy men was published [81]. The bioavailability of DMAU administered on an empty stomach was almost 80 times lower than when a high-fat meal consumption occurred before administration of the substance [81]. When DMAU was administered with food, only 3% of the compound underwent biotransformation to the active form, DMA, which could be detected in serum only 2 h after taking the substance [81]. This effect occurred after taking a dose of 200 mg, 400 mg, or 800 mg [81]. Maximum levels of DMA were detectable in serum 4–6 h after taking DMAU, both on an empty stomach and after a meal. DMA levels in serum remained detectable even 24 h after taking a dose of 400 mg or 800 mg preceded by a high-fat meal [81].

Furthermore, 12 h after oral administration of DMAU with a meal, a dose-dependent decrease in LH and FSH concentrations happened that lasted for 24 h [81]. The amounts of the total as well as free testosterone, estradiol, and dihydrotestosterone were reduced, but this decrease was not dependent on the dose of DMAU [81]. What is more, the study also shows that the single oral doses up to 800 mg of DMAU were not associated with any serious adverse effects (two study participants experienced mild acne) and were well tolerated [81].

A 28-day study thoroughly examined the effects of oral DMAU in a castor oil capsule on men [76]. The substances were administered in doses of 100 mg, 200 mg, and 400 mg. After 7–10 days of regular use of orally administrated DMAU, the endogenous testosterone and gonadotropin secretions were rapidly and strongly suppressed. Moreover, the strength of this inhibition was dose dependent [76]. A dose of 400 mg provided gonadotropin suppression of <1.0 IU/L a threshold level associated with spermatozoid suppression of <1 million/mL [76,82]. The maximum serum concentration of DMAU/DMA was reached after 4 h and the minimum concentration after 24 h, suggesting that the dose taken once a day may be effective [76]. Despite the high testosterone suppression, no significant symptoms of testosterone deficiency were noted. However, there was a significant decrease in libido, weight gain, increased hematocrit, decreased HDL-C, and shortened QTc interval (still within the normal range) [76].

A 2020 study conducted by Brian T. Nguyen et al. investigated the acceptability of DMAU as a male oral hormonal contraceptive among men. The study was conducted as a double-blind trial, in which participants received 1, 2, or 4 capsules containing either a placebo or DMAU (100 mg, 200 mg, 400 mg). Participants were instructed to consume a meal containing 25–30 g of fat prior to taking the drug or placebo [80]. At the end of the study, 82.5% of all participants (both in the experimental and control groups) reported that taking capsules at the same time each day was easy (*p* = 0.03). Nearly all participants (96.5%) agreed that the instructions for capsule intake were clear, while 93% stated that the capsules were easy to swallow and taking them on a full stomach was not problematic. However, 8.8% reported that the capsules had an unpleasant taste and texture, and the need to time food intake within <30 min of administration was an issue. Almost 1/3 of participants (28.1%) reported that they had difficulties adhering to the required fat content in the pre-dose meal due to the excessive amount needed, skipping breakfast, or not knowing how much to consume to meet the requirement. Some stated that consuming 25–30 g of fat for breakfast was too much for them, and they would be unable to take DMAU long-term. Additionally, individuals taking four capsules at a time were more likely to express reluctance to continue therapy compared with those taking two or one capsule (*p* = 0.04) [80].

These findings suggest that the requirement for high-fat meals before DMAU administration and the large number of capsules needed for effective dosing may deter men from adopting this form of contraception. However, the mode of administration and the need for consistent daily dosing did not pose significant challenges. The study also indicates that most participants were satisfied with this contraceptive method and would recommend it to others. If officially available, approximately half of the participants stated they would use DMAU as their primary contraceptive method [80].

The foremost metabolic mechanism leading to low and variable oral bioavailability in men despite oral administration preceded by a fatty meal is a result of the biotransformation of DMA to a major metabolite, glucuronidated 7α,11β-dimethyl-19-nortestosterone (DMA-G), by intestinal and hepatic UDP-glucuronosyltransferase 2B17 (UGT2B17) [83].

DMAU as an oral contraceptive has its drawbacks—patients often do not comply with the daily intake of the drug, and its bioavailability is highly variable and limited. For this reason, an intramuscular form of DMAU with prolonged activity was tested on animals. Cynomolgus monkeys were given the drug once a week for five weeks, but rhesus macaques were injected only once. The observations lasted for over 2 years. After a single injection, DMAU remained in the blood for a week, and its active metabolite, DMA, for over 200 days (1–2 ng/mL). In turn, five injections conducted once a week caused DMA to be present in the blood for over two years. Moreover, the concentration of DMAU increased with the dose, but no such relationship was observed for DMA. A successful depot effect was indicated after intramuscular injection of DMAU, which remained in it while slowly releasing DMA into the bloodstream. The study shows that a single injection in rhesus macaques or five weekly administered injections in cynomolgus macaques led to a decrease in fertility parameters (testosterone, inhibin B, testicular volume, concentration (mln/mL)), spermatozoid morphology, and motility [84].

11β-MNTDC, like DMAU, is administered orally as a prodrug and then is converted to the active form, 11β-methyl-19-nortestosterone (11β-MNT) [77]. 11β-MNT, like DMA, binds to human androgen receptors and progestogen receptors [77]. Administering prodrug with food significantly increases 11β-MNTDC and the concentration of 11β-MNT in serum [77]. However, the conversion of 11β-MNTDC into the active substance is low and ranges from 0.45% (on the first day of administration) to 0.72% (on the second day of administration) during 28 days of use [78].

The concentration of 11β-MNTDC and 11β-MNT is measurable in the blood even after 48 h if the oral dose is equal to 800 mg [77]. A dose-dependent suppression of LH and FSH < 1 IU/L has been demonstrated, allowing for the inhibition of spermatogenesis and a contraceptive effect, which occurs already at 200 mg of oral 11β-MNTDC [78,82]. Suppression of testosterone secretion occurs at a dose of 200 mg. It has been proven that 11β-MNTDC administered at a dose up to 800 mg does not cause serious adverse effects. However, weight gain, acne, and increased or decreased libido may occur [77]. Markers of liver damage remain stable [77,78]. 11β-MNTDC also leads to a minor increase in creatinine levels and a shortening of the QTc interval, but this was not considered clinically significant [78].

In a 28-day study conducted by Brian T. Nguyen et al., the acceptability of 11β-MNTDC as an oral hormonal contraceptive for men was evaluated. The study was designed as a double-blind trial, in which participants received either 2 or 4 pills each day, containing a placebo or 11β-MNTDC (200 mg, 400 mg). Participants were instructed to consume a meal containing 25–30 g of fat before taking the pills. Similar to the findings observed with DMAU [80], most participants reported no difficulty in consistently taking the pills, nor did the act of pill consumption pose a significant issue. However, 15% of the participants stated that taking the pills disrupted their daily routine. One-third of all participants reported experiencing bothersome side effects. Nearly half of the men expressed concerns during the study regarding potential unknown side effects (42.5%), the safety of the pill (27.5%), and its effectiveness (17.5%). Interestingly, 20% of participants reported engaging in sexual activity more frequently while taking the pills, with 18% noting an improvement in their sexual experience [85].

Almost three-quarters of the participants were satisfied with this method of contraception. If it were available for free, 80% of men indicated they would use it as their primary contraceptive method. In contrast, if they had to pay for it, 62.5% reported they would still opt for its use. Additionally, 87.2% of participants stated that this form of contraception met or exceeded their expectations, and nearly all of them would recommend it to a friend. Finally, three-quarters of participants reported that they considered discontinuing pill use no more than once throughout the study [85]. Due to the accessible oral form of 11β-MNTDC administration and a satisfactory treatment regimen (a pill swallowed once a day), the possibility of using this substance as a male hormonal contraception in the future is met with enthusiasm by men [85].

Recent studies showed that DMA, which is a structural equivalent of 11β-MNT, is rapidly glucuronidated by UGT2B17, an enzyme with high genetic and environmental variability [83,86]. Although UGT2B17 is present in the liver, its concentration in the intestine is five times higher. The 11β-MNT is metabolized into two major products—dehydrogenated 11β-MNT and glucuronidated 11β-methyl-19-nortestosterone (11β-MNTG). UGT2B17 is a key enzyme responsible for the formation of 11β-MNTG. The concentration of dehydrogenase enzymes involved in the formation of dehydrogenated 11β-MNT is relatively stable among all cryopreserved human hepatocytes (HHs) used in tests. However, the high genetic variability of the UGT2B17 enzyme among HHs resulted in significantly different amounts of formed 11β-MNTG. This indicates that although both metabolic pathways contribute to the limited bioavailability of prodrugs, UGT2B17 is the main factor responsible for its variability, similar to DMAU [83,86].

UGT2B17 is involved in the metabolism of various other substances, such as gemfibrozil [87], vorinostat [83,86], 17-hydroxyexemestane [83], diclofenac, losartan, valproic acid, thyroxine, and tolcapone [86], which may lead to drug–drug interactions between them and DMA or 11β-MNT. One of the substrates of UGT2B17 is desvenlafaxine, which belongs to the serotonin–noradrenaline reuptake inhibitor (SNRI) class of antidepressants. UDP-glucuronosyltransferase 2B17 enzymes constitute one of the main metabolic pathways for converting desvenlafaxine into inactive metabolites. As a result, co-administering DMA or 11β-MNT with desvenlafaxine could potentially extend the half-life of one of these drugs in the body [83,86,88]. However, certain antidepressants, such as amitriptyline, clomipramine, or imipramine are not metabolized by UGT2B17 and should not cause drug–drug interactions with DMA or 11β-MNT [83,86,89].

Additionally, an in vitro study has shown that warfarin acts as a modifier of glucuronidation reactions. It has been discovered that UGT2B17 contains two binding sites for substances: a catalytic site and an allosteric site. Warfarin binds to the allosteric site, resulting in the inhibition of the glucuronidation reaction [90]. Therefore, it can be hypothesized that the use of an anticoagulant could extend the half-life of DMA and 11β-MNT. However, these are only assumptions, and further research is necessary to precisely determine the interactions between the aforementioned substances.

Neither DMAU/DMA nor 11β-MNTDC/11β-MNT undergo 5α-reduction, unlike testosterone, which is converted to 5α-dihydrotestosterone (5α-DHT) [91]. Consequently, 5α-reduced androgens may have a positive effect on the occurrence of male pattern baldness, acne, or prostate enlargement [91,92]. The lack of 5α-reduction in DMA and 11β-MNT means that they do not display some of the characteristic side effects of anabolic androgens that could be expected after the administration of an exogenous androgen [91,92]. At the same time, the lack of 5α reduction in both of the discussed compounds does not reduce their androgenic potency [91]. Neither DMA nor 11β-MNT are C19 androgens, which makes their aromatization to A-ring aromatic products questionable [93]. The lack of a C19-methyl group causes the aromatization process to proceed slowly or not appear at all [93]. Furthermore, the presence of a methyl group at position 11β, which occurs in both discussed compounds, completely prevents the aromatization process [93]. Aromatization of androgens to A-ring aromatic derivatives is the main route of obtaining estrogens in the human body [93].

Another pathway leading to the formation of compounds with estrogenic activity is the 5α-reduction of 19-norsteroids, which include DMA and 11β-MNT, and the subsequent hydroxylation of the A ring [93]. However, neither DMA nor 11β-MNT undergoes 5α-reduction [91]. This suggests that DMA or 11β-MNT is unlikely to be converted to estrogens in vivo [93].

Testosterone and estrogens increase bone mineral density during puberty [94]. Furthermore, estrogens probably exert a greater influence on the maintenance of bone mass regardless of testosterone [95]. DMA and 11β-MNT, like testosterone, bind to androgen receptors [77,79], but they are not aromatized to estrogens [93]. It is therefore necessary to examine the effect of these compounds on bone density and body composition (percentage of lean mass and fat mass).

A study published in 2013 found that high doses of DMAU or 11β-MNTDC administered subcutaneously to castrated rats for 4 weeks significantly prevented the loss of lean body mass, the gain of fat mass, and the loss of bone mineral density compared with castrated rats that did not receive exogenous androgens [75]. DMAU administration to castrated rats resulted in significantly less fat mass gain when compared with rats that were not castrated. DMAU and 11β-MNTDC significantly increased the weight of the prostate, seminal vesicles, and levator ani muscle compared with castrated rats that did not receive exogenous androgens and rats that were not castrated, with the effects on prostate and seminal vesicle weight being significantly greater with DMAU than with 11β-MNTDC [75].

However, this stimulation is not the desirable effect. The researchers suggest that there should be found a dose of DMAU that maintains bone mineral density and has a beneficial effect on body composition but shows minimal stimulation of the accessory sex glands [75]. The issue of the effect of DMAU on bone turnover was reviewed in men and published in 2021 [94]. A 28-day exposure to the potent androgen DMAU suppressed endogenous testosterone and estradiol production, but it increased the serum marker of bone formation, procollagen type 1 N propeptide (P1NP) [94]. However, no changes were observed in the serum marker of bone resorption, C-terminal telopeptide (CTX), which is an interesting result, considering the lack of aromatization of DMA and the key role of estrogens in maintaining bone mass [94,95].

It is also necessary to determine the effect of DMAU and 11β-MNTDC on human metabolism. Both compounds have been shown to reduce HDL cholesterol as well as SHBG levels and increase hematocrit and body weight to a similar extent after 28 days of oral administration. DMAU causes a decrease in adiponectin, while 11β-MNTDC increases LDL cholesterol. Fasting glucose or insulin levels, as well as the homeostatic model assessment for insulin resistance (HOMA-IR) index, do not change [76,78,96]. One of the side effects of anabolic androgens is potential hepatotoxicity [63]; therefore, it is important to examine the effect of a given substance on the liver before introducing a drug based on it. The need to verify possible hepatotoxicity is all the more important because hormonal contraceptives are used chronically.

A study published in 2010 examined the hepatotoxicity of various androgenic agents that predispose to male hormonal contraception status in adult male rabbits. This work also aimed to verify whether these rodents are a good model for testing the hepatotoxicity of anabolic androgens. The compounds tested included MENT, DMAU, and 11β-MNTDC. Based on the clearance of intravenously injected bromsulfthalein and its retention percentage, it characterized DMAU with the greatest potential of hepatotoxicity. MENT was less hepatotoxic than DMAU, and 11β-MNTDC was the least hepatotoxic compound tested [63]. The results of this study suggest that the C7α methyl group present in DMAU and MENT but absent in 11β-MNTDC may be an important factor in the described hepatotoxicity [63]. Table 5 below provides a summary of the similarities and differences between DMAU and 11β-MNTDC.

## 12. Discussion

Male hormonal contraception offers several advantages, with DMAU (7α,11β-dimethyl-19-nortestosterone 17β-undecanoate) and Nestorone emerging as the most promising options. These methods effectively suppress FSH and LH, reducing sperm production to levels below the contraceptive threshold [65]. DMAU is taken orally [80], making it a convenient option, while Nestorone is applied as a gel [64], offering a non-invasive alternative. Both methods are designed to be fully reversible [66], allowing fertility to return after discontinuation. Additionally, Nestorone does not exhibit androgenic activity [38], which minimizes common side effects such as acne and mood swings. Clinical studies confirm its good safety profile, with only minor side effects like slight weight gain or HDL cholesterol reduction [68,76].

Other hormonal methods also show potential. 11β-MNTDC, an alternative to DMAU, has a longer half-life, requiring fewer doses [77]. MENT (7α-methyl-19-nortestosterone) is another effective agent, but it may increase blood pressure [16]. Overall, male hormonal contraception is advancing toward safe, effective, and reversible options, with DMAU and Nestorone standing out due to their ease of use, minimal side effects, and strong contraceptive efficacy.

Male hormonal contraception creates a new possibility within the range of birth control for men. Its advantages start with the efficacy. First trials of weekly intramuscular injection of 200 mg TE have demonstrated that 98% of men can be suppressed to azoospermia or oligozoospermia. Nevertheless, there have been differences between Asian and non-Asian centers in means of achieved azoospermia [25]. For combination of NES + T, azoospermia occurred in 78% of participants after 8 weeks and 89% after 20–24 weeks [68]. Daily administration of LNG combined with TE injections induced oligospermia in 94% of participants [57]. Similar results in future product studies could challenge the efficacy of condoms. It is harder to predict regarding vasectomy or hormonal methods for women [6,7,12], although on unlike vasectomy, male hormonal contraception is reversible. In surveys, 62.0% and 52.2% of participants were very willing to use the pill and gel, respectively, when compared with a mechanical method, and only 24.2% weighed up the vas-occlusion [97]. According to Gómez-Torres et al. the acceptability of hormonal methods for men was varying times from moderate to high, reaching more than 70% overall [98]. Moreover, the use of male alternatives removes the risk of the significant adverse effects associated with female hormonal contraception, such as thromboembolism, stroke, and myocardial infarction, as well as breast and cervical cancer [99].

A cross-sectional study by Richard et al. with a self-administered questionnaire stated that 87% of the women disagreed with the opinion stating that contraception is only a woman’s affair [100]. Of the women surveyed, 69.7% regarded positively shifting the weight of contraception to men. However, the favor of participants dropped to 46.7% after becoming acquainted with the medical knowledge of the contemporary male contraception methods and those being developed at the time [100]. In the systematic review by Reynolds-Wright, it is stated that male participants of the reviewed studies have been aware of women feeling burdened by contraception [1]. Moreover, the obligation to “take on” the responsibility resulted from the awareness, which could later lead to more equality between the partners [1].

The issue of adverse effects is crucial for safety and the wellbeing of future recipients. At the earliest stages of the search for suitable substances, involving testosterone esters, trials have detected side effects such as symptoms (tenderness, discomfort, pain) at the injection sites, acne, increase in body weight, changes in mood or behavior, self-reported change in libido with predominant increase, blood lipid fluctuations, as well as changes in levels of mean hemoglobin or reduction in total testis volume [18,23,24,25,31]. Although solitary testosterone esters administration trials have been held off, they are still tried in combination with progestins, which can carry similar side effects [45,101].

In a NES + T study [68], in addition to adverse effects like acne (overall 21%), headaches (17%), or changes in sexual function (4%), mood changes were noted. Subjective depressed mood was reported in one, depression in two, altered mood in one, and mood swings in three subjects. Insomnia concerned 6% of subjects.

In a study of the efficacy and safety of intramuscular injections of 200 mg NETE combined with 1000 mg TU [101], there were documented, as possibly related to the method, serious adverse effects, including the following:

- One case of depression during the suppression phase,
- One case of intentional non-fatal paracetamol overdose during the suppression phase,
- One case of tachycardia with paroxysmal atrial fibrillation during the recovery phase.

Cited serious adverse effects raised concerns surrounding mood, which led to more current studies adding validated instruments to better measure mood effects and to inform investigators if these occur [19]. Moreover, 20 of the 320 total participants who received at least 1 injection decided to discontinue due to side effects: 6 men due to changes in mood and another 6 men due to the following single reasons: acne, pain or panic at first injection, palpitations, hypertension, and erectile dysfunction [101]. Eight men discontinued for more than one side effect, of which multiple reasons were associated with changes in mood [101].

A study using the rabbit as a model [63] states that the synthetic androgens 17α-methyltestosterone (MT), DMAU, and MENT resulted in significant increases in percent bone sialoprotein (BSP) dye retention as a marker of hepatotoxicity. However, 11b-MNTDC at a dose of 10 mg/kg/day did not increase the percent BSP dye retention significantly. Oral 11b-MNTDC [77] was well tolerated by healthy men without liver toxicity, with only 2 participants of the study experiencing temporarily increased serum aspartate aminotransferase, alanine aminotransferase, and creatinine kinase concentrations, which was associated with strenuous exercise a few days before. The topic of hepatotoxicity with the intake of male hormonal contraception needs to be investigated more thoroughly on male organisms in a bigger, diverse cohort in a long-term study.

Studies linked to MENT, such as Walton et al., indicate possible minor yet significant increases in systolic blood pressure in participants with MENT implants. The increase may reflect the arterial stiffness, which is inversely related to testosterone levels [54]. Nevertheless, serum levels of testosterone remain generally within the normal range with the most recently developed hormonal male contraceptives [42].

The important mechanisms related to possible cardiovascular risk within male hormonal contraception are associated with, throughout the various studies, increased hematocrit, blood pressure, pro-coagulant substance levels, and generally increased LDL, total cholesterol, and triglycerides, with decreased HDL fraction [18,24,30,31,42,43,45,53,54,68,76,101]. The cause of these fluctuations is based in testosterone’s own impact and progestin’s ability to bind to progesterone and androgen receptors. In the case of the second type, their effect can characterized as anti-androgenic, neutral, or androgenic [42].

In Abbe et al.’s summary of male hormonal contraception’s adverse effects [102], the symptoms reported significantly more by men than women were stressed. The group consisted of injection site pain (23.1%), increased libido (38.1%), night sweats (2.8%), irritability (2.8%), increased appetite (5.0%), hyperhidrosis (5.3%), and musculoskeletal pain and myalgia (20.7%); 5.6% of men reported also gynecomastia. Moreover, male participants reported side effects such as acne, increase in libido, and mood changes more frequently than their female counterparts [102].

Male hormonal contraception exhibits one more side effect connected to its efficacy. Spermatogenic rebound is a phenomenon in which, without a clear reason, the number of sperm cells in semen reaches values above the threshold for effective contraception, even though the contraceptives are used correctly and have been effective up to that point [38]. The aforementioned fact applies to a small proportion of respondents, between 1.4 and 2.2% in the case of substances such as testosterone or testosterone undecanoate administered in the form of injections [18,19,25], while for testosterone in the form of an implant or gel, the incidence of the rebound effect was, respectively, 18% [103] and 7% [104]. This lowers the final assessment of the effectiveness of the tested drugs in preventing pregnancy, and the unpredictability of this phenomenon may be discouraging for men who would like to use hormonal contraceptives.

Challenges in bringing male hormonal contraception to market are also linked to the fact that, unlike women, men do not face the direct risks of unplanned pregnancy and its complications. As a result, the standards for convenience and acceptable side effects in a widely available male contraceptive are higher compared with those applied during the development of hormonal methods for women [48]. Nevertheless, it should also be taken into consideration that some men would be able to accept a certain amount of risk if this would relieve the burden on their partner and consequently reduce their shared risk as a couple [3].

Among the most promising pharmaceuticals tested for suitability for male contraception are undoubtedly DMAU, 11β-MNTDC, and Nestorone. Studies to date indicate the outstanding success of Nestorone combined with testosterone in gel form as a simple-to-use method with negligible adverse effects [68]. However, it is still necessary to assess what effect this agent has on patients during long-term use. The reported slight increase in body weight during use of the test substance appears to be of minor importance in the perspective of the clinical studies to date, but it remains to be investigated whether this symptom suggests a possible increase in the risk of metabolic diseases in long-term use [68,77]. Participants in the study also highlighted the risk of gel transfer during contact with a partner [68], which directs attention toward developing a more convenient form of transdermal application. Phase IIb of the clinical trial to determine the utility of NES + T Combination Gel as a male contraceptive was completed in September 2024 (NCT03452111). However, its results are yet to be published.

Equally promising, DMAU and 11β-MNTDC raise concerns about their significant adverse metabolic effects [76,78,96] and, through their lack of conversion to estrogens, they may also have an effect on bone mass, which would be observable only in a prolonged study [93]. A study of DMAU and 11β-MNTDC in castrated rats strongly suggests such correlation, and its authors point out that a study would need to be conducted to determine the minimum effective dose of these compounds that would result in effective contraception while minimizing adverse effects [73]. Additionally, the risk of hepatotoxic effects of these agents during prolonged use remains unclear [63]. In the case of DMAU, two related studies are listed on ClinicalTrials.gov as of 13 March 2025. The first is a phase I study to determine the safety, pharmacodynamics, and spermatogenesis suppression efficacy of DMAU administered subcutaneously and intramuscularly (NCT02927210). The second one is a phase IIa study directed at assessing the efficacy of inhibiting spermatogenesis of DMAU administered alone or in combination with levonogestrel (NCT03455075). Unfortunately, both of these studies are labeled as studies of unknown status. On 11β-MNTDC, however, several remarkably promising studies have been conducted, showing good tolerability and satisfactory suppression of the orally administered drug [77,78]. However, further studies covering a longer period and a larger number of subjects are needed to assess whether the aforementioned compound will be suitable as a method of safe and reversible hormonal contraception.

Particular attention should be given to the need for studies involving more diverse and larger population groups to better predict the overall effectiveness and safety of male hormonal contraception. A study on an intramuscularly administered testosterone derivative showed that the resulting reduction in sperm production in Caucasians was significantly lower than that in Asians: 70–85% compared with 90–100% [32]. This suggests a strong correlation between the degree of effectiveness of male contraception and ethnicity, which raises the problem that there is still a lack of studies able to provide information on this topic among other races. This problem is aggravated by the under-representation of Black and Hispanic men in studies to date, which may be related to their medical mistrust [105]. This makes a properly extensive assessment of these differences impossible at this moment. This is all the more important given that researchers should be interested in the widest possible trust in the pharmaceuticals introduced, so that they could become an important option for men, including those with financial difficulties [106].

Previous studies dating back to TE alone, like Steinberger et al., documented the reversibility of the male hormonal contraception [23]. According to Meriggiola et al., as the mechanism maintains spermatogonia, the method is supposed to be reversible after discontinuation of the administration [41]. However, the period after which the previous fertility state returned could vary, depending on the study [41]. Data from more recent studies indicated that newer agents like DMAU and 11β-MNTDC in short-time trials, in addition to their initial safety, would also offer reversibility [78,81,107]. Nevertheless, new studies are required, in which long-term trial periods would introduce data assessing the reversibility of the contraceptive after much longer suppression and efficacy phases. Moreover, as the DMAU and 11β-MNTDC studies were conducted on participants in the USA, new multi-center trials ought to inquire into more diverse, intercontinental cohorts [78,81,107].

A crucial research gap linked to newer agents like DMAU and 11β-MNTDC is their future impact on the cardiovascular health of their recipients during long-term administration. A consecutive study of TU indicated changes in cardiovascular risk markers; furthermore, its authors emphasized the need for more extensive testing, including of the aforementioned risk [31]. Similar fluctuations associated with blood morphology and lipid panel were reprised through following studies of different methods like DMPA/DMPA + TE [45] or NES + T [66]. Therefore, studying the cardiovascular safety of the newest agents in the long run should be one of the researchers’ uppermost priorities.

Studies linked to MENT, such as Von Eckardstein et al. [55] and Walton et al. [54], indicate an impact on systolic blood pressure in participants with MENT implants. Von Eckardstein et al. [55] discovered that systolic blood pressure was measurably increased in patients who were administered MENT. Within the first 6 months, mean values increased by 4.8 points. Two of the participants were distinguished by an exceptionally large increase in blood pressure, with initial pressures of 140/90. The first one, during the treatment, had a systolic blood pressure of 150 mm Hg in four out of six scheduled measurements (120 mm Hg and 140 mm Hg in the other two). This resulted in premature discontinuation of the patient. Moreover, the study demonstrated increased (within their normal range) levels of hemoglobin, hematocrit, and erythrocytes. A long-term study from 2007 by Walton et al. [54], subsequently to the aforementioned one, showed a small but consistent increase in systolic blood pressure. The results involved patients administered with either implants of etonogestrel and MENT or etonogestrel implants combined with subcutaneous testosterone pellets. During the study, the first group manifested elevated levels of systolic blood pressure without any significant changes in diastolic blood pressure values. The measurements were higher compared with the second group, with no MENT administered. The treatment furthermore led to a small decrease in HDL cholesterol levels in both groups [54]. The increase in blood pressure could reflect arterial stiffness, which is inversely related to testosterone levels [54]. Nevertheless, serum levels of testosterone remain generally within the normal range with the most recently developed hormonal male contraceptives [42]. Therefore, long-term studies in all new agents are necessary to assess their actual cardiovascular risk and confirm its possible pathomechanisms.

Male hormonal contraception targets several groups of men who may benefit from this method. First, it appeals to those interested in responsible family planning who are willing to share contraceptive responsibility in a heterosexual relationship. Studies indicate that many men are open to using hormonal contraception [2]. Another key group includes partners of women who cannot use traditional hormonal methods due to health risks such as thrombosis or severe side effects [3].

Additionally, male hormonal contraception is an attractive option for individuals seeking a reversible method, as it offers an alternative to vasectomy, which is often considered permanent [9]. It also benefits men who prefer to avoid barrier methods like condoms due to discomfort or inconvenience [7]. Furthermore, research suggests that the effectiveness of hormonal contraception may vary among ethnic groups, highlighting the need for further studies to ensure broad applicability [18]. Lastly, it serves men looking for new contraceptive options beyond condoms and vasectomy [14]. These findings emphasize the growing demand for male hormonal contraception as a safe, effective, and reversible alternative to existing methods.

This study has several limitations. The most significant one is the availability of source materials. Our team used databases provided through affiliation with the Medical University of Wroclaw library. The earliest substances tested for male hormonal contraception were not described in detail, as the review was designed to emphasize the latest research and advancements. This represents another limitation of the present study. Furthermore, the keywords selected for article searches may not have identified all the essential information related to the topic.

## 13. Conclusions

Male hormonal contraception research began in the 1970s, but no drug has replaced barrier or surgical methods. The introduction of progestogens with T-esters improved effectiveness and convenience, with oral forms, patches, and gels offering patient-friendly alternatives despite some skin reactions.

Promising candidates for male hormonal contraception like DMAU and 11β-MNTDC suppress gonadotropin secretion, effectively and reversibly inhibiting sperm production. They are orally bioavailable, do not cause bone loss, and avoid estrogen or 5α-DHT conversion. While 11β-MNTDC has lower hepatotoxicity, both require further study.

Challenges include side effects like acne, weight gain, mood changes, liver concerns, lipid alterations, and cardiovascular risk as well as practical limitations such as frequent application and implant rejection. The rare spermatogenic rebound effect raises reliability concerns.

Advancing male hormonal contraception requires long-term trials to confirm efficacy, safety, and reversibility while minimizing side effects. Future research should encompass broader population groups to more accurately predict the overall efficacy and safety of use. A limitation of many articles is the lack of ethnic diversity in the studies, which should be addressed in future research. Public education, improved drug delivery methods, regulatory approval, and insurance coverage will enhance accessibility. Despite challenges, it remains a desirable, reversible, and non-invasive alternative to vasectomy, promoting shared responsibility in family planning.

## Figures and Tables

**Table 1:** Comparison of available contraceptive methods for men.

Variables	Vasectomy	Condoms
Mode of action	Surgical sterilization	Physical barrier
Prevalence method	2.8% of men worldwide	14% of men worldwide
Failure rate	<1%	13%
Time of action	Constant with three-month delay	Immediate, one-time use
Reversibility	Requires another surgery, which is not always effective	Immediate
Cost and availability	High cost, requires a qualified healthcare provider and a sufficient clinical setting	Low cost, widely available
Protection against sexually transmitted disease	No	Yes
Impact on health	Possible postoperative complications such as hematoma, infection, or chronic testicular pain	Possible issues with erection
Comfort and convenience	Does not affect intercourse	May decrease comfort during intercourse

**Table 2:** Comparative summary chart of testosterone esters.

Variables	TE	TU	TB
Administration	Intramuscular injection	Intramuscular injection	Intramuscular injection
Achieved azoospermia	- 85.7% (Asian centers) - 67.8% (Non-Asian centers)	95.2%	37.5%
Terminal half-life time (in days)	4.5	33.9	29.5
Adverse effects	- Tenderness or discomfort at the injection sites, - Acne, - Body weight increased, - Total cholesterol, LDL, HDL, and triglyceride levels reduced, - Mean hemoglobin increased, - Serum testosterone increased, - Total testis volume reduced, - Serum urea reduced.	- Tenderness or discomfort at the injection sites, - Acne, - Severe coughing lasting minutes after injection, - Changes in mood or behavior, - Facial swelling or skin rash - Self-reported change in libido with predominant increase, - Body weight increased, - Total testis volume reduced, - Mean hemoglobin increased, - Mean total and LDL cholesterol reduced, - Changes in HDL cholesterol levels depending on ethnicity.	- Total testicular volume reduced, - Erythrocyte count increased, - Mean hemoglobin increased, - Hematocrit increased.
Practical limitations	- As a short-acting method, it requires weekly intramuscular injections. - Several months, up to one year, are needed for sperm production to reach a significant suppression. - Injections are the only practically available administration route (common tenderness/discomfort at the injection site).	- Injections are the only practically available administration route (common tenderness/discomfort at the injection site).	- Injections are the only practically available administration route (common tenderness/discomfort at the injection site denied).

TU—testosterone undecanoate; TE—testosterone enanthate; TB—testosterone buciclate; LDL—low-density lipoprotein; HDL—high-density lipoprotein.

**Table 3:** Comparison of gonadotropin suppression in different treatment groups.

Parameter	NES	NES + T
LH and FSH suppression	Moderate/week	Significant
Serum testosterone levels	Decreased	Stable
Libido changes	Decreased	No major decrease
Higher NES dose effect (6–8 mg/day)	Strong gonadotropin suppression	Greater suppression than NES alone
Undetectable LH and FSH levels in subjects	No	Yes

LH—Luteinizing hormone, FSH—follicle-stimulating hormone, NES—nonandrogenic progestin (Nestorone), T—testosterone.

**Table 4:** Comparison of the effectiveness of testosterone (T) and NES progestin therapy at different doses.

Criteria	T + NES 8 mg/Day	T + NES 12 mg/Day
Effectiveness * after 8 weeks	>60%	No improvement compared with 8 mg/day
Effectiveness * after 20–24 weeks	89%	89%
Frequency of azoospermia	78%	69%
Increase in semen volume	Occurred in several men	Occurred in several men

* ≤1 million/mL spermatozoid count. T—testosterone, NES—Nestorone, mg/day—milligrams per day.

**Table 5:** Conformabilities and differences between DMAU and 11β-MNTDC.

Characteristics	DMAU/DMA	11β-MNTDC/11β-MNT
Targeted receptors	Human androgen and progestogen receptors	Human androgen and progestogen receptors
Suppressed hormones	Testosterone, gonadotropins	Testosterone, gonadotropins
Contraceptive spermatogenesis suppression *	Occurs	Occurs
Administrative form	DMAU—prodrug	11β-MNTDC—prodrug
Active form	DMA—transformation happens in body	11β-MNT—transformation happens in body
Route of administration	Oral	Oral
Bioavailability	Greater after high-fat meal	Greater after high-fat meal
Maximal time of detection DMA/11β-MNT (dose 800 mg)	24 h	48 h
Mainly metabolized by	UGT2B17	UGT2B17
5α-reduction	Does not undergo	Does not undergo
Aromatization	Occurs slowly or not at all	Does not occur
Adverse effects	Decreased libido, weight gain, increased hematocrit, decreased HDL cholesterol, decreased adiponectin, decreased SHBG, and shortened QTc interval (within the normal range)	Increased or decreased libido, weight gain, increased hematocrit, acne, increase in creatinine levels, decreased HDL cholesterol, increased LDL cholesterol, decreased SHBG, and shortened QTc interval (within the normal range)
Hepatotoxicity	More toxic	Less toxic

* <1 million/mL spermatozoids in sperm. DMAU—7α,11β-dimethyl-19-nortestosterone 17β-undecanoate, DMA—7α,11β-dimethyl-19-nortestosterone, 11β-MNTDC—11β-methyl-19-nortestosterone 17β-dodecylcarbonate, 11β—MNT-11β-methyl-19-nortestosterone, UGT2B17—UDP-glucuronosyltransferase 2B17, HDL—high-density lipoprotein, LDL—low-density lipoprotein.

## Abbreviations

The following abbreviations are used in this manuscript:

DMAU	7α,11β-dimethyl-19-nortestosterone 17β-undecanoate
11β-MNTDC	11β-methyl-19-nortestosterone 17β-dodecylcarbonate
MENT	7α-methyl-19-nortestosterone
FSH	Follicle-stimulating hormone
LH	Luteinizing hormone
GnRH	Gonadotropin-releasing hormone
HDL	High-density lipoprotein
T	Testosterone
TE	Testosterone enanthate
WHO	World Health Organization
TU	Testosterone undecanoate
TB	Testosterone buciclate
LDL	Low-density lipoprotein
MPA	Medroxyprogesterone acetate
DMPA	Depot medroxyprogesterone acetate
DHT	5α-dihydrotestosterone
EE	Ethinylestradiol
NETE	Norethisterone enanthate
CPA	Cyproterone acetate
LNG	Levonorgestrel
SHBG	Sex hormone-binding globulin
DSG	Desogestrel
MENTAc	7α-methyl-19-nortestosterone acetate
DMA	7α,11β-dimethyl-19-nortestosterone
11β-MNT	11β-methyl-19-nortestosterone
NES	Nestorone
DMA-G	Glucuronidated 7α,11β-dimethyl-19-nortestosterone
UGT2B17	UDP-glucuronosyltransferase 2B17
11β-MNTG	Glucuronidated 11β-methyl-19-nortestosterone
HH	Human hepatocyte
5α-DHT	5α-dihydrotestosterone
SNRI	Serotonin–noradrenaline reuptake inhibitor
P1NP	Procollagen type 1 N propeptide
CTX	C-terminal telopeptide
HOMA-IR	The homeostatic model assessment for insulin resistance
MT	17α-methyltestosterone
BSP	Bone sialoprotein
